# P-1392. A study to evaluate the efficacy, safety, pharmacokinetics-pharmacodynamics of Bedaquiline based regimen in Multibacillary leprosy not responding to WHO-Multidrug therapy (WHO-MDT)

**DOI:** 10.1093/ofid/ofaf695.1579

**Published:** 2026-01-11

**Authors:** Himanshi Khera, Tarun Narang, Seema Chhabra, Debajyoti Chhaterjee, Samir Malhotra, Vimal Kumar Yadav, Abdul Mabood Khan, Kalpana Sahay, Vikas Suri, Sunil Dogra, Nusrat Shafiq

**Affiliations:** Postgraduate Institute of Medical Education and Research (PGIMER), Chandigarh, Chandigarh, Chandigarh, India; PGIMER, Chandigarh, Chandigarh, Chandigarh, India; Postgraduate Institute of Medical Education and Research (PGIMER), Chandigarh, Chandigarh, Chandigarh, India; Postgraduate Institute of Medical Education and Research (PGIMER), Chandigarh, Chandigarh, Chandigarh, India; Postgraduate Institute of Medical Education and Research (PGIMER), Chandigarh, Chandigarh, Chandigarh, India; ICMR- National JALMA Institute of Leprosy & Other Mycobacterial Diseases, Agra, India, Agra, Uttar Pradesh, India; ICMR- National JALMA Institute of Leprosy & Other Mycobacterial Diseases, Agra, India, Agra, Uttar Pradesh, India; ICMR- National JALMA Institute of Leprosy & Other Mycobacterial Diseases, Agra, India, Agra, Uttar Pradesh, India; PGIMER, CHANDIGARH, INDIA, Chandigarh, Chandigarh, India; Postgraduate Institute of Medical Education and Research (PGIMER), Chandigarh, Chandigarh, Chandigarh, India; Postgraduate Institute of Medical Education and Research (PGIMER), Chandigarh, Chandigarh, Chandigarh, India

## Abstract

**Background:**

Leprosy poses a significant challenge in low- and middle-income nations, particularly in certain geographies. While the MDT-MBR regimen has proven beneficial, treatment non-responsiveness remains a key obstacle to eradicating leprosy. Considering bedaquiline's promising outcomes in MDR-TB and pre-clinical leprosy investigations, this proof of concept study aims to ascertain bedaquiline's efficacy and pharmacokinetics-pharmacodynamics in patients with non-responsive multibacillary leprosy.

**Methods:**

The study has two components- preclinical and clinical. Leprosy mouse footpad model was developed by inoculating *M.leprae (* in BALB/C mouse footpad. Groups of animals using different dosing schedules and regimens for bedaquiline are compared against WHO-MDT regimen. Additionally, drug interaction study for evaluating interaction with concomitantly administered drugs was carried out in Swiss albino mice. For clinical study, a prospective, randomized, blinded end point assessment trial comparing bedaquiline based regimen with that of extended WHO-MDT regimen is being undertaken in treatment non responsive patients of leprosy (CTRI Registration No: CTRI/2022/09/045483). Primary and secondary outcomes include key clinical and microbiological criteria.

**Results:**

The preliminary findings of the study are found to be promising. After ensuring *M.leprae* growth in mouse footpad model in mouse after 8 months, it has been found that there is approximately one log decrease in the grown in BALB/C mice after 4 months of treatment with bedaquiline 25 mg/kg once a month. There was no change found in pharmacokinetic profile of bedaquiline, clofazimine and rifampicin when administered altogether as revealed by drug interaction studies. Ten patients have been randomized to each of the treatment arms and are on follow up for observation of outcomes.

**Conclusion:**

In conclusion, the initial findings support the potential of the bedaquiline-based regimen in addressing non-responsive multibacillary leprosy cases with minimal evidence of significant pharmacokinetic drug interaction. The findings warrant further exploration.

**Disclosures:**

All Authors: No reported disclosures

